# Potential for false-positive HIV test results using rapid HIV testing algorithms

**DOI:** 10.4102/ajlm.v4i1.178

**Published:** 2015-09-30

**Authors:** Rosemary A. Audu, Rosemary N. Okoye, Chika K. Onwuamah, Fehintola A. Ige, Adesola Z. Musa, Nkiruka N. Odunukwe, Daniel I. Onwujekwe, Oliver C. Ezechi, Emmanuel O. Idigbe, Phyllis J. Kanki

**Affiliations:** 1 Human Virology Laboratory, Nigerian Institute of Medical Research, Lagos, Nigeria; 2 Clinical Diagnostic Laboratory, Nigerian Institute of Medical Research, Lagos, Nigeria; 3 Monitoring and Evaluation Unit, Nigerian Institute of Medical Research, Lagos, Nigeria; 4 Clinical Sciences Division, Nigerian Institute of Medical Research, Lagos, Nigeria; 5 Harvard School of Public Health, Boston, Massachusetts, United States

## Abstract

**Background:**

In order to scale up access to HIV counselling and testing in Nigeria, an HIV diagnostic algorithm based on rapid testing was adopted. However, there was the need to further evaluate the testing strategy in order to better assess its performance, because of the potential for false positivity.

**Objectives:**

The objective of this study was to compare positive HIV test results obtained from the approved rapid testing algorithm with results from western blot tests performed on samples from the same patient.

**Methodology:**

A retrospective review was conducted of HIV screening and confirmatory results for patients seen between 2007 and 2008. Rapid test and western blot results were extracted and compared for concordance. Discordant results were further reviewed using a combination of HIV-1 RNA viral load and CD4+ cell count test results and clinical presentation from medical records.

**Results:**

Analysis of 2228 western blot results showed that 98.3% (*n* = 2191) were positive for HIV-1, 0.4% (*n* = 8) were positive for HIV-2 and 0.3% (*n* = 7) were dual infections (positive for both HIV-1 and HIV-2); 0.6% (*n* = 13) were indeterminate and 0.4% (*n* = 9) were negative. Further investigation of the 13 indeterminate results showed nine to be HIV-1 positive and four to be HIV-negative, for a total of 13 negative results. The positive predictive value of the HIV counselling and testing algorithm was 99.4%.

**Conclusion:**

Using the rapid testing algorithm alone, false positives were detected. Therefore, effective measures such as training and retraining of staff should be prioritised in order to minimise false-positive diagnoses and the associated potential for long-term psychological and financial impact on the patients.

## Introduction

The diagnosis of HIV infection is most often based on the detection of serum antibodies to HIV. These serological tests could be classified either as screening tests, such as rapid tests or enzyme linked immuno-sorbent assay (ELISA), or confirmatory tests, such as western blot (WB). Prior to the availability of rapid testing, same-day results were not obtainable and an estimated one third of individuals tested did not return to learn their HIV status.^1^ Compared with ELISA and WB, rapid test assays are cheaper, easier to perform and the results are readily available on the same day. In 1999, the World Health Organization and the Joint United Nations Programme on HIV/AIDS recommended the use of a combination of rapid test assays or ELISAs to confirm positive results, employing a highly sensitive test as the first screening test and a second, highly specific confirmatory test to verify the detection of antibodies specific to HIV. This was considered to be as reliable for confirmation as WB but at a much lower cost.^2^

In 2007, the government of Nigeria adopted a serial testing algorithm with three combinations of the following rapid test assays: Determine® (Abbott, Tokyo, Japan), Stat-Pak® (Chembio Diagnostic Systems, Medford, New York, United States) or Uni-Gold™ (Trinity Biotech, Jamestown, New York, United States). Samples with discordant results were further tested with Bundi RT (BUNDI International Diagnostics Ltd, Aba, Nigeria) as the tiebreaker.^3^ The adopted algorithm could be used in any of the combinations shown in Table 1. Because of quality issues with the Bundi test kits, the use of the kit was suspended; the third combination was retained and is currently in use in the country.

**TABLE 1:** Algorithm adopted in 2007 for HIV rapid testing in Nigeria.

Screening test	Confirmation of positives	Tie-breaker
Determine	Stat-Pak	Bundi
Uni-Gold	Stat-Pak	Bundi
Determine	Uni-Gold	Stat-Pak

As with all screening assays, HIV rapid test screening has a certain degree of false-positive results, irrespective of the algorithm in use.^4^ For this reason, a confirmatory test is necessary for a definitive diagnosis. Evaluations of HIV rapid testing strategies have been reported in some African countries where the results have been used to formulate alternative HIV testing strategies.^5,6^ Regardless of which rapid test algorithm is used, false-positive results may still occur;^7,8^ 1% – 2% of reactive rapid test results have been found to be negative with an HIV nucleic acid test.^7^ Since the introduction of the 2007 HIV rapid test strategy in Nigeria, its performance has not been evaluated. Therefore, the assessment of the algorithm’s performance remains an important priority to better inform national policy and ensure accurate HIV diagnosis and surveillance. The aim of this study was to compare positive HIV test results obtained from the approved rapid testing algorithm with results from WB tests performed on samples from the same patient.

## Methods

### Study setting

The study was conducted at the Nigerian Institute of Medical Research (NIMR), Lagos, a parastatal of the Federal Ministry of Health. NIMR has an HIV treatment centre that currently provides comprehensive HIV care, antiretroviral treatment (ART) and support for over 18 000 patients. The centre is supported by the Government of Nigeria and the US President’s Emergency Plan for AIDS Relief-funded programme for ART; as a result, all services are provided free to patients. Patients who screen positive for HIV at the HIV Counselling and Testing (HCT) centre or any government of Nigeria-approved HIV counselling and testing centre are referred to the treatment programme for further management. These patients are clinically assessed and sent to the NIMR virology laboratory for baseline laboratory assessment. The NIMR virology laboratory is an ISO 9001:2008 certified laboratory and employs well trained and competent personnel to perform the laboratory assays. Quality assurance measures undertaken by the laboratory include: participation in external quality assessment for all assays; equipment maintenance; and several measures to verify pre-analytical, analytical and post-analytical processes.

### National testing algorithm

The approved national serial testing algorithm used for this study was: Determine, Unigold and Stat-Pak as tie breaker. This algorithm is currently in use in Nigeria; thus, the results of this study are relevant to the current situation in the country.

### Study design

This was a retrospective study, which analysed data from the programme’s electronic database. Cases with discordant results were further reviewed using a combination of plasma viral load, CD4+ cell counts and clinical data to determine a presumptive HIV diagnosis or progression of infection. HIV test results generated between January 2007 and August 2008 were analysed. Discordant results were reviewed until December 2011.

### Data abstraction and inclusion criteria

Data were abstracted from records of patients aged 18 years or older who had tested positive for HIV using the 2007 Nigerian rapid test algorithm at the NIMR HCT, satellite sites or private clinics. Data for patients sent to the NIMR virology laboratory for confirmation of HIV diagnosis by WB were abstracted for this study. Data from all patients who had given informed consent to participate in research studies were included. WB test result data included all positive, negative and indeterminate results. Indeterminate results were defined as reactivity profiles that were not compatible with either a positive or a negative interpretation.

### Laboratory methods

The NIMR HCT site conducted HIV testing using the national testing algorithm; this required the use of non-cold chain dependent rapid test kits supplied by the government of Nigeria through the Central Medical Stores. The satellite sites and private clinics who referred HIV-positive patients for care and treatment also indicated the rapid test kits used in obtaining the positive HIV results. At the NIMR virology laboratory, the baseline assessment included: HIV confirmation using the WB technique (Immunetics Inc., Boston, Massachusetts, United States) to detect specific viral proteins for both HIV-1 and HIV-2; CD4+ cell count (cells/µL) using the flow cytometry technique employed by the Partec Cyflow Counter II (Gorlitz, Germany) to determine eligibility for the initiation of ART; and quantitation of HIV-1 RNA viral load in plasma (Roche Amplicor v1.5, Mannheim, Germany) with a lower limit of detection of 400 copies/mL. The viral load assay was further used to monitor the effectiveness of therapy and for timely identification of poor adherence and possible virologic failure.

### Statistical analyses

The positive predictive value was calculated based on the results of the rapid tests and WB using standard formulae. Exact 95% confidence intervals for these proportions were calculated. Analyses were conducted using Statistical Package for Social Sciences version 17 for Windows (IBM Corporation, Chicago, Illinois, United States).

### Ethical considerations

Ethical approval for this study was obtained from the NIMR and Harvard School of Public Health Institutional Review Boards. Written informed consent (dated 14 November 2006) was obtained prior to enrolment of patients for both services and research.

## Results

Results from 2228 different patients meeting the inclusion criteria were extracted from the programme’s electronic database. There was a male: female ratio of 1.9:1 and the mean age was 38.8 ± 9.0 years. The NIMR HCT facility accounted for 967 (43.4%) of the results abstracted, whereas 1261 (56.6%) were from satellite sites and private clinics. The analyses of the WB results showed that 98.3% were positive for HIV-1, 0.4% were positive for HIV-2 and 0.3% had HIV-1 and HIV-2 dual infections. There were also 13 (0.6%) indeterminate and nine (0.4%) negative results (Table 2). The 13 indeterminate results were further investigated using plasma viral load levels and nine (69.2%) were found to be infected with HIV-1 based on very high HIV-1 viral load levels (median = 310 377 RNA copies/mL; range = 2150–991 706 RNA copies/mL), whereas four (30.8%) were considered to be HIV-negative based on undetectable viral load results (<400 RNA copies/mL). Thus, a total of 13 results that had been positive according to the rapid test algorithm were actually negative. This yielded a false-positive rate of 0.6%.

**TABLE 2:** HIV-positive rapid test results obtained by western blot technique, NIMR, January 2007 − August 2008.

HIV Status	Number	Percentage
HIV-1	2191	98.3
HIV-2	8	0.4
HIV-1 and HIV-2	7	0.3
Indeterminate	13†	0.6
Negative	9	0.4
Total	2228	100

NIMR, Nigerian Institute of Medical Research.

†, Further investigation by viral load revealed nine were HIV-1 positive and four HIV-negative, resulting in a total of 13 (0.6%) HIV false positives.

Clinical records of cases with negative WB results were reviewed. After a minimum of 12 months with no clinical evidence of infection or laboratory evidence of HIV disease, these cases were discontinued from the clinic. The four cases with indeterminate WB results and undetectable viral load results were monitored for an additional two years. In the absence of any clinical manifestation of HIV infection and repeat undetectable viral load results, they were discharged from the clinic. Taking both the true positives and false positives into consideration, the positive predictive value was calculated to be 99.4% (99.0% – 99.6% CI). The satellite sites and private clinics were responsible for 46.2% (6/13) of the false-positive results. Upon further investigation, we found that all patients at these facilities (100%) had been screened using Determine and Stat-Pak test kits, including those with false-positive results. Since both kits produced concordant results, a third kit was not required as a tie breaker for any of these cases. The immunological and virological data of these false positives were analysed and it was found that their median CD4+ cell count was 668 cells/µL (range = 50–1500 cells/µL) and all had an undetectable viral load (< 400 RNA copies/mL).

## Discussion

Rapid HIV antibody tests help to improve access to testing in both clinical and nonclinical settings, as well as increasing the proportion of people who receive their results once tested. As good as the rapid HIV screening assays may be, a confirmatory test is still required for a definitive diagnosis because of the possibility of false-positive results. False positives may result from numerous causes, including the inherent characteristics of the assay, or may be because of the genetic diversity of the virus.^4^ The percentage of false positivity reported in this study was 0.6%, which is similar to that reported in other studies,^3,9^ but is lower than the 2% reported in Cameroon.^10^ As part of Nigeria’s global AIDS response progress report in 2012, 11.7% of the population of 162 265 000 had received an HIV test within the last 12 months and knew their HIV status.^11^ This corresponds to 18 985 005 tests performed in Nigeria within the year. Given the false-positive rate of 0.6% found in this study, approximately 113 910 people per year who are not HIV-positive receive a false HIV-positive diagnosis. The US President’s Emergency Plan for AIDS Relief reported the yearly cost of management of an HIV patient to be US $338.^12^ These 113 910 false-positive diagnoses could result in US $38.5M per year in inappropriate but significant costs for HIV management. These resources could be better used for the estimated 1 512 720 HIV-infected individuals still requiring ART.

Possible ways to identify patients with false-positive results include: lack of risk factors for HIV, normal CD4+ cell count and undetectable viral load.^13^ In Nigeria, the CD4+ cell count reference value has been reported to be 365–1571 cells/µL.^14^ Causes of false-positive results include: fictitious HIV exposure/infection^15^ and HIV vaccination,^16^ the latter being unlikely because HIV vaccine trials have not been conducted in Nigeria. Other likely reasons for false-positive results could be either clerical or technical. The possibility of these errors in the NIMR virology reference laboratory is low because of the laboratory’s stringent quality management system. None of the laboratories that carried out the HCT for the results included in this study had implemented a quality management system. This is not unusual, considering the dearth of knowledge about quality management systems in most laboratories in Africa, including Nigeria.^17^ The inconsistent or incorrect use of the rapid test algorithm has also been reported as a possible cause of false-positive results.^18^ In addition, false-positive results could also be a result of poor algorithm planning or implementation.^19^ The incorrect interpretation of weak-positive test lines and non-usage of tie-breaker algorithms, which occurs in our setting, could explain the latter.

The use of confirmatory WB allowed for the diagnosis of HIV-1 and HIV-2, between which these particular rapid test assays cannot discriminate. As seen in previous studies,^20^ HIV-1 was the major HIV type amongst patients accessing HIV care in our clinic. The very low rate of HIV-2 and HIV-1/2 dual infections observed in this study could be attributed to the decline in its prevalence, as reported in other West African countries,^21^ resulting in part from the inefficiency of transmission of HIV-2.^22^ In this study, men had a higher uptake of testing than women. This is similar to observations made between 2003 and 2007 (about the same timeframe as this study), but by 2011, this finding had been reversed.^11^ This is most likely attributable to the increased availability of prevention of mother-to-child transmission treatment, as well as treatment including paediatric therapy.

The delivery of a false-positive HIV result to a patient can be devastating, as it often results in psychological stress and trauma, particularly with subsequent stigmatisation. In a study from three African countries, namely, the Democratic Republic of Congo, Burundi and Ethiopia, some patients who were falsely diagnosed as HIV-positive had been abandoned by their partners after they started on ART or prophylaxis.^23^ There are also substantial financial costs related to misdiagnosis, where patients might lose time and compensation from work in order to attend clinic visits. In addition, the possible side effects and toxicities of unnecessary ART need to be considered. In our study, patients with false-positive results were found to have high median CD4+ cell counts and undetectable viral loads. Neither of these conditions can rule out the possibility of HIV infection, particularly if patients are natural elite controllers^24^ or receiving ART. It has also been reported that there are apparently healthy Nigerians who have CD4+ cell counts below 350 cells/µL.^17,25^

It is important to note that the cost of side effects, psychological trauma and lifelong treatment is unquantifiable. There have been reports of suicides resulting from a diagnosis of HIV,^26^ hence the gravity of false-positive results should be well-recognised. It is therefore essential that great care and caution is exercised in HIV diagnosis.

In spite of the challenge of false-positive results, the use of rapid test kits doubled the proportion of individuals who had been tested and received their test results between 2003 and 2007.^11^ The calculated positive predictive value of 99.4% in this study is similar to the established national positive predictive value of 99.5% (96.8% – 99.9% CI) based on the national algorithm’s sensitivity of 100% and specificity of 99.7%.^3^ This implies that the use of this algorithm is still very essential in the expansion of HIV testing in a highly-populated country such as Nigeria, if the spread of this virus is to be controlled.

### Limitations

This study is, however, not without limitations. The sensitivity, specificity and negative predictive values could not be estimated, because only HIV-positive patients were referred to the treatment centre for further follow up, including the WB re-testing. In like manner, not all cases referred from the HCT centres eventually reported to the treatment centre. It is also important to note that although WB is a confirmatory test, it does have a window period of six weeks; thus, its usefulness for the detection of acute infections is limited,^27,28^ as there is a possibility of missing early infections. Although rapid test kits also have a window period, the higher sensitivity assay is used initially, then followed by a very specific test. The long follow up of patients with discordant test results and repeat testing should have reduced the effect of the six-week window period. The WB assay also generates some indeterminate results. However, an indeterminate result is still acceptable in comparison to a false-positive result, as it allows for further evaluation.^29^

### Conclusion

In this study, we found a 0.6% rate of HIV false positives with the implementation of the 2007 Nigerian HIV rapid testing algorithm. It is therefore recommended that clinicians should refer suspicious false-positive patients for WB confirmation. All possible measures should be taken to prevent false-positive diagnoses associated with rapid testing alone, as the long-term costs of treatment, side effects and psychological trauma to patients may be considerable.
